# Crystal structure of *rac*-(3a’*R*,9a’*R*)-3a’-(indol-3-yl)-1′,2′,3′,3a’,4′,9a’-hexa­hydro­spiro­[cyclo­pentane-1,9′-penta­leno[1,2-*b*]indole] *p*-xylene hemisolvate

**DOI:** 10.1107/S2056989015007422

**Published:** 2015-04-18

**Authors:** Wayland E. Noland, Matthew A. Worth, Andrew K. Schneerer, Courtney L. Paal, Kenneth J. Tritch

**Affiliations:** a Department of Chemistry, University of Minnesota, Minneapolis, MN 55455-0431, USA

**Keywords:** crystal structure, annulation, indole, cyclic ketone, disorder, *X*—H⋯π inter­actions

## Abstract

The title compound, C_26_H_26_N_2_·0.5C_8_H_10_, is the first reported characterized 2:2 product from acid-catalyzed condensation of indole with cyclo­penta­none and no other 2:2 products were observed. Recrystallization from *p*-xylene gave the title hemisolvate with the *p*-xylene mol­ecule located about an inversion center. The terminal penta­lene ring is envelope-flap disordered at the C atom farthest from the skeletal indole unit, with a refined occupancy ratio of 0.819 (4):0.181 (4). The major component has this C atom bent away from the *spiro*-fused cyclo­pentane ring. In the crystal, mol­ecules are connected by N—H⋯π inter­actions, forming chains along [100], and N—H⋯π and C—H⋯π inter­actions, forming chains along [001], which results in the formation of slabs parallel to (010).

## Abstract 2

The title compound is the first reported characterized 2:2 product from acid-catalyzed condensation of indole with cyclo­penta­none. In the crystal, mol­ecules are connected by a series of N—H⋯π and C—H⋯π inter­actions, forming slabs parallel to (010).

## Chemical context   

**Figure 1:**
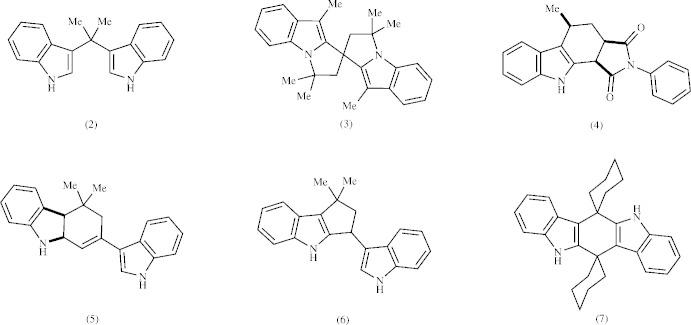
Contextual compounds.

Condensations of indole with ketones and aldehydes under mildly acidic conditions generally give 2:1 bis­indole products analogous to (2) (see Fig. 1 ▸; Shiri *et al.*, 2010 ▸). Several examples have shown anti­cancer activity (Maciejewska *et al.*, 2006 ▸; Lee *et al.*, 2008 ▸), although biological activities are more commonly observed from bis­indoles that include additional heterocyclic moieties (Gu *et al.*, 1999 ▸; Andreani *et al.*, 2008 ▸). Strong acid catalysts, such as BF_3_ etherate, give higher-order products, including (3) (Banerji *et al.*, 1983 ▸). Moderate conditions, such as dilute hydro­chloric acid, generally favor 2:2 products. When a good dienophile is present, the inter­mediate 3-vinyl­indole can be trapped by a Diels–Alder reaction, giving cyclo­adducts such as (4) (Noland *et al.*, 1993 ▸).

**Figure 2:**
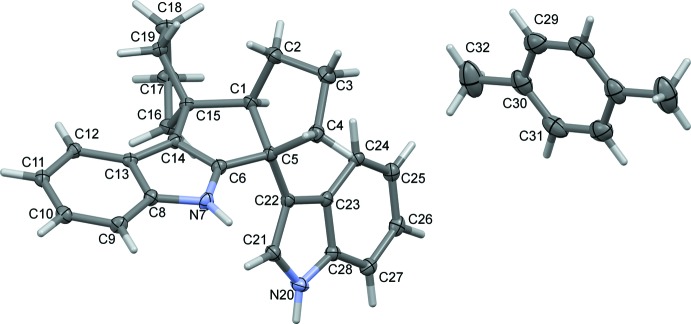
The mol­ecular structure of the title compound, showing the atom labeling. For clarity, only the major component is shown. Displacement ellipsoids are drawn at the 50% probability level. Unlabeled xylene atoms are related by the symmetry code −*x* + 2, −*y*, −*z* + 2.

For most ketones and aldehydes, the major 2:2 product is of type (5) or (6) (Bergman *et al.*, 1989 ▸). A noteworthy exception is the cyclo­hexa­none product (7), reported by Guzei *et al.* (2012 ▸), which exhibits inter­esting physical and fluorescence properties. It was desirable to explore the use of cyclic ketones of other sizes to determine whether analogs of (7) might be obtained. To date, the only observed 2:2 products have been analogs of the title compound, (1) (Fig. 2 ▸). These products lack the physical and fluorescence behaviors shown by (7) and have been obtained as powders or crystalline solvates.
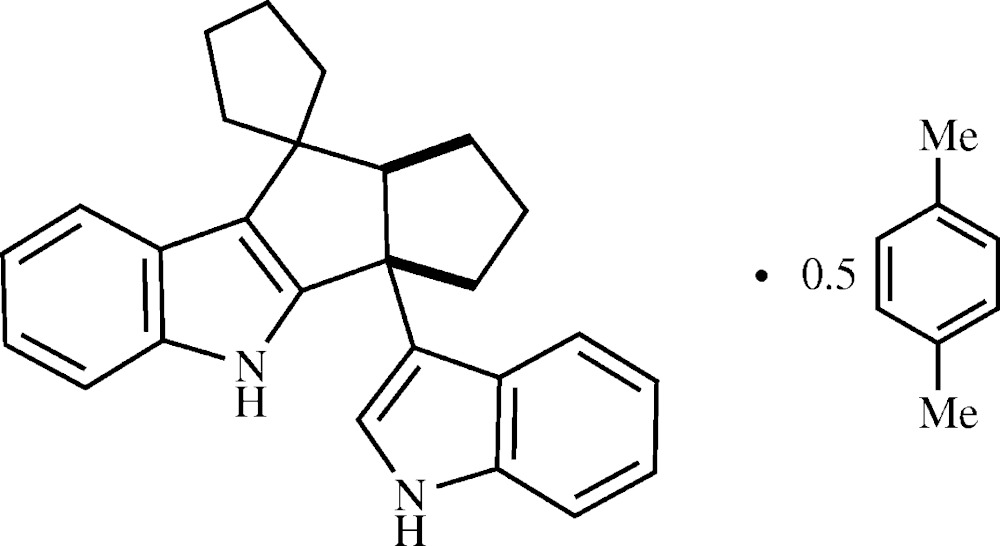


## Structural commentary   

**Figure 3:**
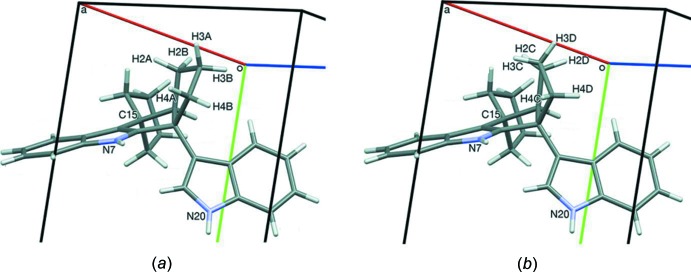
The (*a*) major and (*b*) minor components of compound (1) in the crystal, viewed roughly along [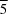
0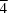]. The H atoms attached to the atoms that change position (*viz*. C2, C3, and C4) are labeled. Note the envelope conformation of the C15–C19 ring.

The indole units are inclined to one another by 63.85 (4)° and are nearly planar, with r.m.s. deviations from their mean planes of 0.013 and 0.007 Å for C8–C13/C6/N7 and N20/C21–C28, respectively. The C1–C5 ring is C3/C3′ flap-disordered in two twist–envelope conformers, with a refined occupancy ratio of 0.819 (4):0.181 (4) (Fig. 3 ▸). The C15–C19 ring adopts an envelope conformation bent away from atom H12, with atom C15 as the flap.

## Supra­molecular features   

**Figure 4:**
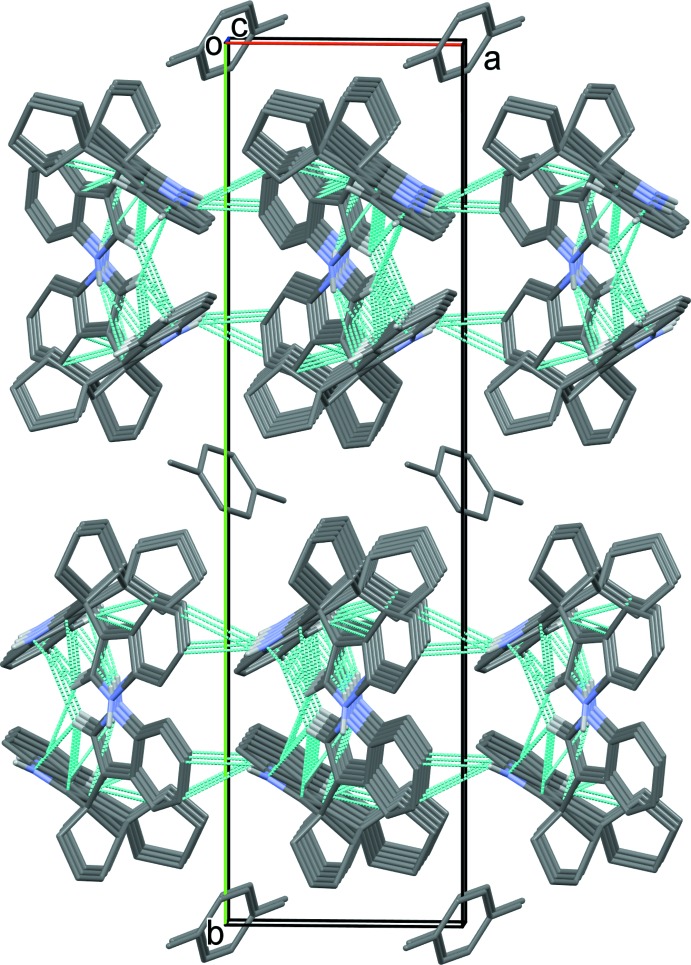
The crystal packing of compound (1), viewed along the *c* axis. Only the H atoms involved in the various inter­molecular contacts have been included (see Table 1 ▸ for details).

For lack of classical hydrogen-bond acceptors, it was anti­cipated that one or both N—H hydrogens would form short contacts with a ring centroid of another indole unit. Two N—H⋯π contacts are present; however, the axes of both N—H donor bonds are oblique and exocyclic to the acceptor rings. These and several C—H⋯π contacts are summarized in Table 1 ▸. The H7⋯*Cg*2 distance is *ca* 3.185 Å, too large to be considered a classical H⋯*Cg* contact. Therefore, atom H7 is depicted as forming a non-classical hydrogen bond with atom C26, the nearest C atom. Hence, the N7—H7⋯C26 contacts form chains along [100]. The distance of this contact, *ca* 2.66 Å, can be compared with the generic C⋯H van der Waals distance of 2.88 Å reported by Rowland & Taylor (1996 ▸). The various C—H⋯*Cg* contacts and the N20—H20⋯*Cg*3 contact form chains along [001]; see Table 1 ▸. The combination of these various contact leads to the formation of slabs parallel to (001). Glide planes are surrounded by indole systems, whereas inversion centers border the aliphatic portions of (1) and *p*-xylene (Fig. 4 ▸). Although crystals of (1) were only obtained as a solvate, there are no short contacts between (1) and *p*-xylene.

## Database survey   

**Figure 5:**
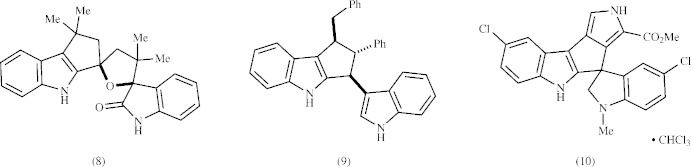
Database survey entries.

A search of the Cambridge Structural Database (Version 5.36; update of November 2014; Groom & Allen, 2014 ▸) found several entries that are synthetically or structurally related to (1). Compound (8) formed *via* 2:3 condensation of indole with acetone, and autoxidation (Banerji *et al.*, 1981 ▸; Fig. 1 ▸). Compound (9), prepared by ZnBr_2_-catalyzed cyclo­dimerization of *trans*-3-(β-styr­yl)indole, features a pendant indol-3-yl group in the same position as (1) and similar but stronger N—H⋯π contacts in the crystal (McNulty & McLeod, 2011 ▸; Fig. 5 ▸). No entries were found that contain the penta­leno[1,2-*b*]indole functionality, although (10) has a skeleton similar to (1) (Zhang *et al.*, 2012 ▸).

## Synthesis and crystallization   

Indole (1.17 g) was dissolved in cyclo­penta­none (10 ml). After the system was flushed with nitro­gen, concentrated hydro­chloric acid (0.1 ml) was added. The resulting mixture was heated to 350 K for 5 d. After cooling to room temperature, di­chloro­methane (DCM, 20 ml), water (20 ml), sodium bicarbonate (500 mg), and sodium bis­ulfite solution (saturated, 30 ml) were added. The resulting mixture was stirred for 2 h. The organic portion was filtered through neutral alumina (H = 2 cm × D = 3 cm; DCM), and then concentrated at reduced pressure. The resulting residue was separated by column chromatography (SiO_2_, hexa­ne–ethyl acetate, gradient from 1:0 to 5:1). The desired fraction (*R_f_* = 0.43 in 2:1) was concentrated at reduced pressure, giving the title compound as a white powder (yield: 877 mg, 48%; m.p. 466–468 K); ^1^H NMR (500 MHz, CD_2_Cl_2_): δ 8.005 (*s*, 1H, H20), 7.921 (*s*, 1H, H7), 7.518 (*d*, *J* = 7.0 Hz, 1H, H12), 7.480 (*d*, *J* = 7.9 Hz, 1H, H24), 7.354 (*d*, *J* = 7.9 Hz, 1H, H27), 7.320 (*d*, *J* = 7.1 Hz, 1H, H9), 7.146 (*dd*, *J* = 7.9, 7.7 Hz, 1H, H26), 7.081 (*td*, *J* = 7.1, 1.6, 1H, H10), 7.049 (*td*, *J* = 7.0, 1.6, 1H, H11), 7.018 (*dd*, *J* = 7.9, 7.7 Hz, 1H, H25), 6.806 (*d*, *J* = 2.6 Hz, 1H, H21), 3.090 (*dd*, *J* = 8.3, 5.6 Hz, 1H, H1), 2.500 (*dt*, *J* = 13.0, 7.3 Hz, 1H, H4*B*/*D*), 2.235 (*dt*, *J* = 13.0, 6.6 Hz, 1H, H4*A*/*C*), 2.165 (*dt*, *J* = 13.0, 8.7 Hz, 1H, H19*A*), 2.085–1.820 (*m*, 6H, H2*B*/*D*, H3*B*/*D*, H18*A*, H19*B*, H2*A*/*C*, H16*A*), 1.793–1.693 (*m*, 3H, H17*A*, H18*B*, H3*A*/*C*), 1.612–1.547 (*m*, 1H, H17*B*), 1.500 (*ddd*, *J* = 11.9, 7.3, 4.0 Hz, 1H, H16*B*); ^13^C NMR (126 MHz, CD_2_Cl_2_): δ 146.98 (C6), 141.67 (C8), 137.77 (C28), 126.29 (C23), 125.12 (C14), 124.36 (C13), 123.69 (C22), 122.26 (C26), 121.86 (C21), 120.99 (C10), 120.59 (C24), 119.75 (C11), 119.65 (C25), 118.99 (C12), 112.30 (C9), 111.18 (C27), 68.48 (C1), 54.26 (C5), 53.41 (C15), 42.34 (C16), 39.00 (C4), 33.72 (C19), 31.43 (C2), 28.08 (C3), 25.16 (C17, C18); IR (KBr, cm^−1^) 3413 (*vs*, N—H), 3044 (*w*), 2953 (*s*), 2868 (C—H), 1446 (*s*, C=C), 1250, 1101 (C—N), 1015, 749 (*s*, C—H); MS (EI, *m*/*z*) [*M*]^+^ calculated for C_26_H_26_N_2_ 366.21, found 366.21. Analysis (Atlantic Microlab, Norcross, GA, USA) calculated for C_26_H_26_N_2_: C 85.21, H 7.15, N 7.64%; found C 85.30, H 7.18, N 7.62%.

Recrystallization was attempted from common solvents. The best crystals were obtained from *p*-xylene. Attempted sublimation (0.012 mm Hg, 460 K) of neat or hemisolvate samples resulted in slow decomposition with elimination of indole. The sublimate was a light yellow powder, roughly 93 mol% compound (1). No useful sublimed crystals were found.

## Refinement   

Crystal data, data collection, and structure refinement details are summarized in Table 2 ▸. H atoms were placed in calculated positions and refined as riding atoms, with N—H = 0.88 Å and C—H = 0.95–1.00 Å, and with *U*_iso_(H) = 1.5*U*_eq_(C) for methyl H atoms and 1.2*U*_eq_(N,C) for other H atoms. The C1–C5 ring is disordered over two components with a refined occupancy ratio of 0.819 (4):0.181 (4). The disordered components were refined such that the only atoms occupying different sites are C3/C3′ and H atoms riding on C2/C2′, C3/C3′, and C4/C4′.

## Supplementary Material

Crystal structure: contains datablock(s) I, global. DOI: 10.1107/S2056989015007422/su5116sup1.cif

Structure factors: contains datablock(s) I. DOI: 10.1107/S2056989015007422/su5116Isup2.hkl

Supporting information file. DOI: 10.1107/S2056989015007422/su5116Isup3.cml

CCDC reference: 1059822

Additional supporting information:  crystallographic information; 3D view; checkCIF report

## Figures and Tables

**Table 1:** Hydrogen-bond geometry (, ) *Cg*1, *Cg*2, *Cg*3 and *Cg*4 are the centroids of rings N20/C21C23/C28, C23C28, C6/N7/C8/C13/C14 and C8C13, respectively.

*D*H*A*	*D*H	H*A*	*D* *A*	*D*H*A*
N7H7C26^i^	0.88	2.66	3.493(2)	157
C11H11*Cg*1^ii^	0.95	2.82	3.5419(16)	133
C12H12*Cg*2^ii^	0.95	2.70	3.4652(16)	138
N20H20*Cg*3^iii^	0.88	2.82	3.5654(14)	144
C21H21*Cg*4^iii^	0.95	2.92	3.4970(17)	120

Symmetry codes: (i) 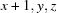; (ii) 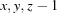; (iii) 
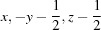
.

**Table 2:** Experimental details

Crystal data	
Chemical formula	C_26_H_26_N_2_0.5C_8_H_10_
*M*_r_	419.57
Crystal system, space group	Monoclinic, *P*2_1_/*c*
Temperature (K)	173
*a*, *b*, *c* ()	8.7618(7), 29.450(2), 9.6569(8)
()	114.732(1)
*V* (^3^)	2263.2(3)
*Z*	4
Radiation type	Mo *K*
(mm^1^)	0.07
Crystal size (mm)	0.35 0.21 0.13

Data collection	
Diffractometer	Bruker APEXII CCD
Absorption correction	Multi-scan (*SADABS*; Sheldrick, 1996 ▸)
*T*_min_, *T*_max_	0.700, 0.746
No. of measured, independent and observed [*I* > 2(*I*)] reflections	25883, 5184, 4102
*R*_int_	0.032

Refinement	
*R*[*F* ^2^ > 2(*F* ^2^)], *wR*(*F* ^2^), *S*	0.048, 0.126, 1.06
No. of reflections	5184
No. of parameters	294
No. of restraints	321
H-atom treatment	H-atom parameters constrained
_max_, _min_ (e ^3^)	0.51, 0.37

Computer programs: *APEX2* and *SAINT* (Bruker, 2007 ▸), *SHELXS97* (Sheldrick, 2008 ▸), *SHELXL2014* (Sheldrick, 2015 ▸), *Mercury* (Macrae *et al.*, 2008 ▸), *enCIFer* (Allen *et al.*, 2004 ▸), and *publCIF* (Westrip, 2010 ▸).

## supplementary crystallographic information

### Crystal data

**Table:**

C_26_H_26_N_2_·0.5C_8_H_10_	*D*_x_ = 1.231 Mg m^−^^3^
*M**_r_* = 419.57	Melting point: 459 K
Monoclinic, *P*2_1_/*c*	Mo *K*α radiation, λ = 0.71073 Å
*a* = 8.7618 (7) Å	Cell parameters from 2946 reflections
*b* = 29.450 (2) Å	θ = 2.4–27.3°
*c* = 9.6569 (8) Å	µ = 0.07 mm^−^^1^
β = 114.732 (1)°	*T* = 173 K
*V* = 2263.2 (3) Å^3^	Block, colourless
*Z* = 4	0.35 × 0.21 × 0.13 mm
*F*(000) = 900	

### Data collection

**Table:**

Bruker APEXII CCD diffractometer	4102 reflections with *I* > 2σ(*I*)
Radiation source: sealed tube	*R*_int_ = 0.032
φ and ω scans	θ_max_ = 27.5°, θ_min_ = 2.4°
Absorption correction: multi-scan (*SADABS*; Sheldrick, 1996)	*h* = −11→11
*T*_min_ = 0.700, *T*_max_ = 0.746	*k* = −38→38
25883 measured reflections	*l* = −12→12
5184 independent reflections	

### Refinement

**Table:**

Refinement on *F*^2^	321 restraints
Least-squares matrix: full	Hydrogen site location: inferred from neighbouring sites
*R*[*F*^2^ > 2σ(*F*^2^)] = 0.048	H-atom parameters constrained
*wR*(*F*^2^) = 0.126	*w* = 1/[σ^2^(*F*_o_^2^) + (0.0542*P*)^2^ + 0.8656*P*] where *P* = (*F*_o_^2^ + 2*F*_c_^2^)/3
*S* = 1.06	(Δ/σ)_max_ < 0.001
5184 reflections	Δρ_max_ = 0.51 e Å^−^^3^
294 parameters	Δρ_min_ = −0.37 e Å^−^^3^

### Special details

**Table:**

Geometry. All e.s.d.'s (except the e.s.d. in the dihedral angle between two l.s. planes) are estimated using the full covariance matrix. The cell e.s.d.'s are taken into account individually in the estimation of e.s.d.'s in distances, angles and torsion angles; correlations between e.s.d.'s in cell parameters are only used when they are defined by crystal symmetry. An approximate (isotropic) treatment of cell e.s.d.'s is used for estimating e.s.d.'s involving l.s. planes.

### Fractional atomic coordinates and isotropic or equivalent isotropic displacement parameters (Å^2^)

**Table:**

	*x*	*y*	*z*	*U*_iso_*/*U*_eq_	Occ. (<1)
C1	0.44662 (19)	0.09927 (5)	0.06623 (16)	0.0282 (3)	
H1	0.3383	0.1017	0.0771	0.034*	
C2	0.5227 (3)	0.05186 (5)	0.1165 (2)	0.0462 (4)	0.819 (4)
H2A	0.5987	0.0441	0.0678	0.055*	0.819 (4)
H2B	0.4328	0.0287	0.0876	0.055*	0.819 (4)
C2'	0.5227 (3)	0.05186 (5)	0.1165 (2)	0.0462 (4)	0.181 (4)
H2C	0.5252	0.0353	0.0284	0.055*	0.181 (4)
H2D	0.4529	0.0343	0.1561	0.055*	0.181 (4)
C3	0.6179 (3)	0.05373 (7)	0.2856 (2)	0.0428 (5)	0.819 (4)
H3A	0.7056	0.0299	0.3217	0.051*	0.819 (4)
H3B	0.5417	0.0496	0.3367	0.051*	0.819 (4)
C3'	0.6949 (6)	0.0567 (2)	0.2367 (9)	0.0428 (5)	0.181 (4)
H3C	0.7774	0.0576	0.1913	0.051*	0.181 (4)
H3D	0.7229	0.0309	0.3089	0.051*	0.181 (4)
C4	0.6968 (2)	0.10088 (5)	0.31725 (17)	0.0335 (3)	0.819 (4)
H4A	0.8117	0.0998	0.3215	0.040*	0.819 (4)
H4B	0.7039	0.1125	0.4160	0.040*	0.819 (4)
C4'	0.6968 (2)	0.10088 (5)	0.31725 (17)	0.0335 (3)	0.181 (4)
H4C	0.8122	0.1133	0.3680	0.040*	0.181 (4)
H4D	0.6504	0.0968	0.3939	0.040*	0.181 (4)
C5	0.58207 (17)	0.13233 (5)	0.18429 (15)	0.0261 (3)	
C6	0.66437 (17)	0.14891 (5)	0.08495 (16)	0.0262 (3)	
N7	0.80742 (15)	0.17360 (4)	0.11129 (13)	0.0292 (3)	
H7	0.8814	0.1831	0.2005	0.035*	
C8	0.81381 (17)	0.18073 (5)	−0.02801 (16)	0.0260 (3)	
C9	0.93197 (17)	0.20408 (5)	−0.06175 (17)	0.0292 (3)	
H9	1.0285	0.2173	0.0165	0.035*	
C10	0.90408 (18)	0.20741 (5)	−0.21329 (17)	0.0299 (3)	
H10	0.9825	0.2234	−0.2395	0.036*	
C11	0.76282 (18)	0.18777 (5)	−0.32891 (16)	0.0284 (3)	
H11	0.7467	0.1908	−0.4321	0.034*	
C12	0.64618 (17)	0.16398 (5)	−0.29538 (16)	0.0253 (3)	
H12	0.5512	0.1505	−0.3746	0.030*	
C13	0.67016 (16)	0.16009 (4)	−0.14320 (15)	0.0230 (3)	
C14	0.57911 (17)	0.13989 (5)	−0.06520 (15)	0.0240 (3)	
C15	0.41925 (17)	0.11434 (4)	−0.09859 (15)	0.0241 (3)	
C16	0.26029 (17)	0.14373 (5)	−0.17142 (17)	0.0297 (3)	
H16A	0.2674	0.1639	−0.2506	0.036*	
H16B	0.2454	0.1627	−0.0934	0.036*	
C17	0.11381 (19)	0.11002 (5)	−0.2425 (2)	0.0371 (4)	
H17A	0.0327	0.1209	−0.3435	0.044*	
H17B	0.0542	0.1062	−0.1759	0.044*	
C18	0.1935 (2)	0.06478 (5)	−0.2582 (2)	0.0382 (4)	
H18A	0.1389	0.0533	−0.3642	0.046*	
H18B	0.1822	0.0416	−0.1889	0.046*	
C19	0.37848 (18)	0.07557 (5)	−0.21480 (16)	0.0282 (3)	
H19A	0.3959	0.0853	−0.3053	0.034*	
H19B	0.4499	0.0487	−0.1689	0.034*	
N20	0.46954 (16)	0.24080 (4)	0.31417 (15)	0.0329 (3)	
H20	0.4794	0.2703	0.3303	0.039*	
C21	0.55349 (19)	0.21573 (5)	0.24806 (17)	0.0308 (3)	
H21	0.6315	0.2278	0.2129	0.037*	
C22	0.50921 (17)	0.17099 (5)	0.23994 (15)	0.0250 (3)	
C23	0.38834 (17)	0.16807 (5)	0.30492 (15)	0.0245 (3)	
C24	0.29604 (18)	0.13280 (5)	0.33118 (16)	0.0281 (3)	
H24	0.3062	0.1026	0.3015	0.034*	
C25	0.19025 (18)	0.14242 (5)	0.40062 (17)	0.0320 (3)	
H25	0.1275	0.1186	0.4183	0.038*	
C26	0.17390 (19)	0.18680 (6)	0.44541 (17)	0.0335 (3)	
H26	0.0999	0.1925	0.4925	0.040*	
C27	0.26304 (18)	0.22218 (5)	0.42229 (17)	0.0321 (3)	
H27	0.2531	0.2522	0.4540	0.039*	
C28	0.36823 (18)	0.21257 (5)	0.35091 (16)	0.0277 (3)	
C29	0.9782 (3)	−0.02919 (8)	0.8839 (3)	0.0599 (5)	
H29	0.9643	−0.0498	0.8039	0.072*	
C30	0.8857 (3)	0.01069 (8)	0.8504 (3)	0.0570 (5)	
C31	0.9101 (3)	0.03952 (7)	0.9693 (3)	0.0593 (6)	
H31	0.8488	0.0672	0.9502	0.071*	
C32	0.7658 (3)	0.02275 (12)	0.6900 (3)	0.0962 (10)	
H32A	0.7930	0.0530	0.6647	0.144*	
H32B	0.6505	0.0226	0.6819	0.144*	
H32C	0.7760	0.0004	0.6190	0.144*	

### Atomic displacement parameters (Å^2^)

**Table:**

	*U*^11^	*U*^22^	*U*^33^	*U*^12^	*U*^13^	*U*^23^
C1	0.0335 (8)	0.0263 (7)	0.0256 (7)	−0.0062 (6)	0.0131 (6)	−0.0011 (5)
C2	0.0684 (12)	0.0257 (8)	0.0365 (9)	−0.0030 (8)	0.0141 (8)	0.0047 (7)
C3	0.0532 (13)	0.0311 (10)	0.0395 (11)	0.0073 (9)	0.0150 (9)	0.0062 (8)
C4	0.0364 (8)	0.0354 (8)	0.0275 (7)	0.0046 (6)	0.0121 (6)	0.0042 (6)
C2'	0.0684 (12)	0.0257 (8)	0.0365 (9)	−0.0030 (8)	0.0141 (8)	0.0047 (7)
C3'	0.0532 (13)	0.0311 (10)	0.0395 (11)	0.0073 (9)	0.0150 (9)	0.0062 (8)
C4'	0.0364 (8)	0.0354 (8)	0.0275 (7)	0.0046 (6)	0.0121 (6)	0.0042 (6)
C5	0.0276 (7)	0.0277 (7)	0.0219 (6)	−0.0033 (5)	0.0092 (5)	0.0003 (5)
C6	0.0244 (7)	0.0276 (7)	0.0253 (7)	−0.0020 (5)	0.0090 (5)	−0.0002 (5)
N7	0.0248 (6)	0.0383 (7)	0.0209 (6)	−0.0074 (5)	0.0061 (5)	−0.0033 (5)
C8	0.0235 (7)	0.0280 (7)	0.0244 (7)	0.0007 (5)	0.0081 (5)	0.0002 (5)
C9	0.0217 (7)	0.0335 (8)	0.0307 (7)	−0.0033 (6)	0.0091 (6)	−0.0021 (6)
C10	0.0257 (7)	0.0316 (7)	0.0353 (8)	0.0004 (6)	0.0157 (6)	0.0027 (6)
C11	0.0299 (7)	0.0309 (7)	0.0257 (7)	0.0032 (6)	0.0128 (6)	0.0024 (6)
C12	0.0239 (7)	0.0261 (7)	0.0241 (7)	0.0009 (5)	0.0083 (5)	−0.0008 (5)
C13	0.0208 (6)	0.0216 (6)	0.0255 (7)	0.0018 (5)	0.0086 (5)	0.0001 (5)
C14	0.0243 (7)	0.0229 (6)	0.0238 (7)	0.0006 (5)	0.0089 (5)	0.0003 (5)
C15	0.0254 (7)	0.0220 (6)	0.0242 (6)	−0.0022 (5)	0.0097 (5)	−0.0013 (5)
C16	0.0270 (7)	0.0252 (7)	0.0352 (8)	−0.0001 (5)	0.0114 (6)	0.0004 (6)
C17	0.0257 (8)	0.0368 (8)	0.0448 (9)	−0.0045 (6)	0.0110 (7)	−0.0022 (7)
C18	0.0337 (8)	0.0326 (8)	0.0446 (9)	−0.0087 (6)	0.0127 (7)	−0.0072 (7)
C19	0.0308 (7)	0.0252 (7)	0.0276 (7)	−0.0037 (6)	0.0111 (6)	−0.0037 (5)
N20	0.0377 (7)	0.0210 (6)	0.0374 (7)	−0.0013 (5)	0.0132 (6)	0.0010 (5)
C21	0.0318 (8)	0.0288 (7)	0.0303 (7)	−0.0030 (6)	0.0115 (6)	0.0036 (6)
C22	0.0263 (7)	0.0263 (7)	0.0200 (6)	−0.0027 (5)	0.0073 (5)	0.0017 (5)
C23	0.0245 (7)	0.0257 (7)	0.0192 (6)	−0.0004 (5)	0.0050 (5)	0.0011 (5)
C24	0.0282 (7)	0.0281 (7)	0.0259 (7)	−0.0031 (6)	0.0092 (6)	−0.0014 (5)
C25	0.0270 (7)	0.0387 (8)	0.0289 (7)	−0.0028 (6)	0.0104 (6)	0.0026 (6)
C26	0.0261 (7)	0.0452 (9)	0.0266 (7)	0.0063 (6)	0.0086 (6)	0.0002 (6)
C27	0.0298 (7)	0.0322 (8)	0.0280 (7)	0.0076 (6)	0.0058 (6)	−0.0014 (6)
C28	0.0269 (7)	0.0259 (7)	0.0243 (7)	0.0014 (5)	0.0046 (6)	0.0018 (5)
C29	0.0650 (13)	0.0548 (12)	0.0731 (15)	−0.0183 (10)	0.0420 (12)	−0.0085 (10)
C30	0.0449 (11)	0.0646 (13)	0.0655 (13)	−0.0117 (9)	0.0270 (10)	0.0147 (10)
C31	0.0563 (12)	0.0447 (11)	0.0901 (16)	0.0027 (9)	0.0436 (12)	0.0157 (11)
C32	0.0633 (15)	0.139 (3)	0.0742 (17)	−0.0256 (16)	0.0172 (13)	0.0335 (17)

### Geometric parameters (Å, º)

**Table:**

C1—C2'	1.536 (2)	C14—C15	1.5013 (18)
C1—C2	1.536 (2)	C15—C16	1.5376 (19)
C1—C5	1.5886 (19)	C15—C19	1.5347 (19)
C1—C15	1.5711 (19)	C16—C17	1.539 (2)
C1—H1	1.0000	C16—H16A	0.9900
C2—C3	1.492 (3)	C16—H16B	0.9900
C2—H2A	0.9900	C17—C18	1.541 (2)
C2—H2B	0.9900	C17—H17A	0.9900
C2'—C3'	1.476 (4)	C17—H17B	0.9900
C2'—H2C	0.9900	C18—C19	1.528 (2)
C2'—H2D	0.9900	C18—H18A	0.9900
C3—C4	1.524 (2)	C18—H18B	0.9900
C3—H3A	0.9900	C19—H19A	0.9900
C3—H3B	0.9900	C19—H19B	0.9900
C3'—C4'	1.513 (4)	N20—C21	1.374 (2)
C3'—H3C	0.9900	N20—C28	1.3670 (19)
C3'—H3D	0.9900	N20—H20	0.8800
C4—C5	1.561 (2)	C21—C22	1.366 (2)
C4—H4A	0.9900	C21—H21	0.9500
C4—H4B	0.9900	C22—C23	1.4418 (19)
C4'—C5	1.561 (2)	C23—C24	1.4032 (19)
C4'—H4C	0.9900	C23—C28	1.4182 (19)
C4'—H4D	0.9900	C24—C25	1.382 (2)
C5—C6	1.5025 (19)	C24—H24	0.9500
C5—C22	1.510 (2)	C25—C26	1.403 (2)
C6—N7	1.3775 (18)	C25—H25	0.9500
C6—C14	1.3509 (19)	C26—C27	1.375 (2)
N7—C8	1.3856 (18)	C26—H26	0.9500
N7—H7	0.8800	C27—C28	1.392 (2)
C8—C9	1.390 (2)	C27—H27	0.9500
C8—C13	1.4211 (19)	C29—C30	1.386 (3)
C9—C10	1.383 (2)	C29—C31^i^	1.378 (3)
C9—H9	0.9500	C29—H29	0.9500
C10—C11	1.399 (2)	C30—C31	1.372 (3)
C10—H10	0.9500	C30—C32	1.505 (3)
C11—C12	1.385 (2)	C31—C29^i^	1.378 (3)
C11—H11	0.9500	C31—H31	0.9500
C12—C13	1.3995 (19)	C32—H32A	0.9800
C12—H12	0.9500	C32—H32B	0.9800
C13—C14	1.4356 (19)	C32—H32C	0.9800
			
C2—C1—C15	116.07 (12)	C12—C13—C14	135.45 (13)
C2—C1—C5	103.60 (12)	C6—C14—C13	107.61 (12)
C2'—C1—C5	103.60 (12)	C6—C14—C15	112.11 (12)
C2'—C1—C15	116.07 (12)	C13—C14—C15	140.21 (12)
C5—C1—C15	107.79 (11)	C1—C15—C14	100.99 (11)
C2—C1—H1	109.7	C1—C15—C16	110.34 (11)
C5—C1—H1	109.7	C1—C15—C19	114.89 (11)
C15—C1—H1	109.7	C14—C15—C16	113.66 (11)
C1—C2—C3	106.41 (14)	C14—C15—C19	116.15 (11)
C1—C2—H2A	110.4	C16—C15—C19	101.26 (11)
C1—C2—H2B	110.4	C15—C16—C17	105.53 (11)
C3—C2—H2A	110.4	C15—C16—H16A	110.6
C3—C2—H2B	110.4	C17—C16—H16A	110.6
H2A—C2—H2B	108.6	C15—C16—H16B	110.6
C1—C2'—C3'	109.1 (3)	C17—C16—H16B	110.6
C1—C2'—H2C	109.9	H16A—C16—H16B	108.8
C1—C2'—H2D	109.9	C16—C17—C18	105.90 (12)
C3'—C2'—H2C	109.9	C16—C17—H17A	110.6
C3'—C2'—H2D	109.9	C16—C17—H17B	110.6
H2C—C2'—H2D	108.3	C18—C17—H17A	110.6
C2—C3—C4	104.62 (15)	C18—C17—H17B	110.6
C2—C3—H3A	110.8	H17A—C17—H17B	108.7
C2—C3—H3B	110.8	C17—C18—C19	105.40 (12)
C4—C3—H3A	110.8	C17—C18—H18A	110.7
C4—C3—H3B	110.8	C17—C18—H18B	110.7
H3A—C3—H3B	108.9	C19—C18—H18A	110.7
C2'—C3'—C4'	105.9 (2)	C19—C18—H18B	110.7
C2'—C3'—H3C	110.5	H18A—C18—H18B	108.8
C2'—C3'—H3D	110.5	C15—C19—C18	104.54 (12)
C4'—C3'—H3C	110.5	C15—C19—H19A	110.8
C4'—C3'—H3D	110.5	C15—C19—H19B	110.8
H3C—C3'—H3D	108.7	C18—C19—H19A	110.8
C3—C4—C5	107.06 (13)	C18—C19—H19B	110.8
C3—C4—H4A	110.3	H19A—C19—H19B	108.9
C3—C4—H4B	110.3	C21—N20—C28	109.01 (12)
C5—C4—H4A	110.3	C21—N20—H20	125.5
C5—C4—H4B	110.3	C28—N20—H20	125.5
H4A—C4—H4B	108.6	N20—C21—C22	110.53 (13)
C3'—C4'—C5	102.9 (3)	N20—C21—H21	124.7
C3'—C4'—H4C	111.2	C22—C21—H21	124.7
C3'—C4'—H4D	111.2	C5—C22—C21	126.53 (13)
C5—C4'—H4C	111.2	C5—C22—C23	127.37 (12)
C5—C4'—H4D	111.2	C21—C22—C23	105.97 (13)
H4C—C4'—H4D	109.1	C22—C23—C24	134.96 (13)
C1—C5—C4	104.92 (11)	C22—C23—C28	106.94 (12)
C1—C5—C4'	104.92 (11)	C24—C23—C28	118.10 (13)
C1—C5—C6	98.91 (11)	C23—C24—C25	119.36 (14)
C1—C5—C22	114.67 (11)	C23—C24—H24	120.3
C4—C5—C6	113.69 (12)	C25—C24—H24	120.3
C4'—C5—C6	113.69 (12)	C24—C25—C26	121.09 (14)
C4—C5—C22	112.17 (11)	C24—C25—H25	119.5
C4'—C5—C22	112.17 (11)	C26—C25—H25	119.5
C6—C5—C22	111.68 (11)	C25—C26—C27	121.17 (14)
C5—C6—N7	134.14 (12)	C25—C26—H26	119.4
C5—C6—C14	115.07 (12)	C27—C26—H26	119.4
N7—C6—C14	110.72 (12)	C26—C27—C28	117.73 (14)
C6—N7—C8	107.70 (11)	C26—C27—H27	121.1
C6—N7—H7	126.2	C28—C27—H27	121.1
C8—N7—H7	126.2	N20—C28—C23	107.55 (12)
N7—C8—C9	129.71 (13)	N20—C28—C27	129.90 (14)
N7—C8—C13	108.23 (12)	C23—C28—C27	122.54 (13)
C9—C8—C13	122.05 (13)	C30^i^—C29—C31	121.5 (2)
C8—C9—C10	117.64 (13)	C30—C29—H29	119.3
C8—C9—H9	121.2	C31^i^—C29—H29	119.3
C10—C9—H9	121.2	C29—C30—C31	117.2 (2)
C9—C10—C11	121.40 (13)	C29—C30—C32	121.9 (2)
C9—C10—H10	119.3	C31—C30—C32	120.9 (2)
C11—C10—H10	119.3	C29—C31—C30^i^	121.3 (2)
C10—C11—C12	121.05 (13)	C29^i^—C31—H31	119.3
C10—C11—H11	119.5	C30—C31—H31	119.3
C12—C11—H11	119.5	C30—C32—H32A	109.5
C11—C12—C13	119.06 (13)	C30—C32—H32B	109.5
C11—C12—H12	120.5	C30—C32—H32C	109.5
C13—C12—H12	120.5	H32A—C32—H32B	109.5
C8—C13—C12	118.80 (12)	H32A—C32—H32C	109.5
C8—C13—C14	105.74 (12)	H32B—C32—H32C	109.5
			
C5—C1—C2—C3	32.92 (18)	C6—N7—C8—C13	0.36 (16)
C15—C1—C2—C3	150.85 (15)	N7—C8—C9—C10	−177.64 (14)
C5—C1—C2'—C3'	−7.4 (4)	C13—C8—C9—C10	1.1 (2)
C15—C1—C2'—C3'	110.5 (4)	N7—C8—C13—C12	178.11 (12)
C2—C1—C5—C4	−15.51 (15)	N7—C8—C13—C14	−0.65 (15)
C2—C1—C5—C6	102.06 (13)	C9—C8—C13—C12	−0.9 (2)
C2—C1—C5—C22	−139.03 (13)	C9—C8—C13—C14	−179.63 (13)
C2'—C1—C5—C4'	−15.51 (15)	C8—C9—C10—C11	−0.5 (2)
C2'—C1—C5—C6	102.06 (13)	C9—C10—C11—C12	−0.4 (2)
C2'—C1—C5—C22	−139.03 (13)	C10—C11—C12—C13	0.6 (2)
C15—C1—C5—C4	−139.04 (12)	C11—C12—C13—C8	0.0 (2)
C15—C1—C5—C4'	−139.04 (12)	C11—C12—C13—C14	178.27 (14)
C15—C1—C5—C6	−21.48 (14)	C8—C13—C14—C6	0.70 (15)
C15—C1—C5—C22	97.44 (13)	C8—C13—C14—C15	177.17 (16)
C2—C1—C15—C14	−94.01 (15)	C12—C13—C14—C6	−177.75 (15)
C2—C1—C15—C16	145.46 (14)	C12—C13—C14—C15	−1.3 (3)
C2—C1—C15—C19	31.79 (18)	C6—C14—C15—C1	−13.29 (15)
C2'—C1—C15—C14	−94.01 (15)	C6—C14—C15—C16	104.85 (14)
C2'—C1—C15—C16	145.46 (14)	C6—C14—C15—C19	−138.25 (13)
C2'—C1—C15—C19	31.79 (18)	C13—C14—C15—C1	170.34 (16)
C5—C1—C15—C14	21.58 (14)	C13—C14—C15—C16	−71.5 (2)
C5—C1—C15—C16	−98.95 (13)	C13—C14—C15—C19	45.4 (2)
C5—C1—C15—C19	147.39 (12)	C1—C15—C16—C17	−85.04 (14)
C1—C2—C3—C4	−37.5 (2)	C14—C15—C16—C17	162.36 (12)
C1—C2'—C3'—C4'	28.4 (7)	C19—C15—C16—C17	37.06 (14)
C2—C3—C4—C5	26.9 (2)	C1—C15—C19—C18	77.39 (15)
C2'—C3'—C4'—C5	−37.3 (6)	C14—C15—C19—C18	−165.10 (12)
C3—C4—C5—C1	−6.50 (17)	C16—C15—C19—C18	−41.49 (14)
C3—C4—C5—C6	−113.49 (15)	C15—C16—C17—C18	−18.99 (16)
C3—C4—C5—C22	118.60 (15)	C16—C17—C18—C19	−6.91 (17)
C3'—C4'—C5—C1	32.1 (4)	C17—C18—C19—C15	30.33 (16)
C3'—C4'—C5—C6	−74.9 (4)	C28—N20—C21—C22	0.09 (17)
C3'—C4'—C5—C22	157.2 (4)	C21—N20—C28—C23	−0.48 (16)
C1—C5—C6—N7	−169.36 (16)	C21—N20—C28—C27	178.45 (15)
C1—C5—C6—C14	14.02 (15)	N20—C21—C22—C5	−175.78 (13)
C4—C5—C6—N7	−58.7 (2)	N20—C21—C22—C23	0.33 (16)
C4—C5—C6—C14	124.72 (14)	C5—C22—C23—C24	−3.6 (3)
C4'—C5—C6—N7	−58.7 (2)	C5—C22—C23—C28	175.45 (13)
C4'—C5—C6—C14	124.72 (14)	C21—C22—C23—C24	−179.71 (15)
C22—C5—C6—N7	69.5 (2)	C21—C22—C23—C28	−0.61 (15)
C22—C5—C6—C14	−107.11 (14)	C22—C23—C24—C25	178.81 (15)
C1—C5—C22—C21	−132.41 (15)	C28—C23—C24—C25	−0.2 (2)
C1—C5—C22—C23	52.30 (18)	C22—C23—C28—N20	0.67 (15)
C4—C5—C22—C21	108.04 (16)	C22—C23—C28—C27	−178.36 (13)
C4—C5—C22—C23	−67.25 (18)	C24—C23—C28—N20	179.96 (12)
C4'—C5—C22—C21	108.04 (16)	C24—C23—C28—C27	0.9 (2)
C4'—C5—C22—C23	−67.25 (18)	C23—C24—C25—C26	−0.1 (2)
C6—C5—C22—C21	−20.9 (2)	C24—C25—C26—C27	−0.3 (2)
C6—C5—C22—C23	163.78 (13)	C25—C26—C27—C28	0.9 (2)
C5—C6—N7—C8	−176.63 (15)	C26—C27—C28—N20	179.94 (14)
C14—C6—N7—C8	0.10 (16)	C26—C27—C28—C23	−1.3 (2)
C5—C6—C14—C13	176.90 (11)	C31^i^—C29—C30—C31	0.1 (3)
C5—C6—C14—C15	−0.66 (17)	C31^i^—C29—C30—C32	178.78 (19)
N7—C6—C14—C13	−0.51 (16)	C29—C30—C31—C29^i^	−0.1 (3)
N7—C6—C14—C15	−178.07 (12)	C32—C30—C31—C29^i^	−178.80 (19)
C6—N7—C8—C9	179.23 (15)		

Symmetry code: (i) −*x*+2, −*y*, −*z*+2.

### Hydrogen-bond geometry (Å, º)

*Cg*1, *Cg*2, *Cg*3 and *Cg*4 are the centroids of rings N20/C21–C23/C28, C23–C28, C6/N7/C8/C13/C14 and C8–C13, respectively.

**Table:**

*D*—H···*A*	*D*—H	H···*A*	*D*···*A*	*D*—H···*A*
N7—H7···C26^ii^	0.88	2.66	3.493 (2)	157
C11—H11···*Cg*1^iii^	0.95	2.82	3.5419 (16)	133
C12—H12···*Cg*2^iii^	0.95	2.70	3.4652 (16)	138
N20—H20···*Cg*3^iv^	0.88	2.82	3.5654 (14)	144
C21—H21···*Cg*4^iv^	0.95	2.92	3.4970 (17)	120

Symmetry codes: (ii) *x*+1, *y*, *z*; (iii) *x*, *y*, *z*−1; (iv) *x*, −*y*−1/2, *z*−1/2.

## References

[bb1] Allen, F. H., Johnson, O., Shields, G. P., Smith, B. R. & Towler, M. (2004). *J. Appl. Cryst.* **37**, 335–338.

[bb2] Andreani, A., Burnelli, S., Granaiola, M., Leoni, A., Locatelli, A., Morigi, R., Rambaldi, M., Varoli, L., Landi, L., Prata, C., Berridge, M. V., Grasso, C., Fiebig, H.-H., Kelter, G., Burger, A. M. & Kunkel, M. W. (2008). *J. Med. Chem.* **51**, 4563–4570. 10.1021/jm800194k PMC2754042 18598018

[bb3] Banerji, J., Chatterjee, A., Manna, S., Pascard, C., Prange, T. & Shoolery, J. N. (1981). *Heterocycles*, **15**, 325–336.

[bb4] Banerji, J., Mustafi, R. & Shoolery, J. N. (1983). *Heterocycles*, **20**, 1355–1362.

[bb5] Bergman, J., Norrby, P.-O., Tilstam, U. & Venemalm, L. (1989). *Tetrahedron*, **45**, 5549–5564.

[bb6] Bruker (2007). *APEX2* and *SAINT*. Bruker AXS Inc., Madison, Wisconsin, USA.

[bb7] Groom, C. R. & Allen, F. H. (2014). *Angew. Chem. Int. Ed.* **53**, 662–671. 10.1002/anie.201306438 24382699

[bb8] Gu, X.-H., Wan, X.-Z. & Jiang, B. (1999). *Bioorg. Med. Chem. Lett.* **9**, 569–572. 10.1016/s0960-894x(99)00037-2 10098665

[bb9] Guzei, I. A., Spencer, L. C., Codner, E. & Boehm, J. M. (2012). *Acta Cryst.* E**68**, o1–o2. 10.1107/S1600536811051208 PMC3254282 22259386

[bb10] Lee, C.-H., Yao, C.-F., Huang, S.-M., Ko, S., Tan, Y.-H., Lee-Chen, G.-J. & Wang, Y.-C. (2008). *Cancer*, **113**, 815–825. 10.1002/cncr.23619 18618576

[bb11] Maciejewska, D., Szpakowska, I., Wolska, I., Niemyjska, M., Mascini, M. & Maj-Żurawska, M. (2006). *Bioelectrochemistry*, **69**, 1–9. 10.1016/j.bioelechem.2005.09.003 16307909

[bb12] Macrae, C. F., Bruno, I. J., Chisholm, J. A., Edgington, P. R., McCabe, P., Pidcock, E., Rodriguez-Monge, L., Taylor, R., van de Streek, J. & Wood, P. A. (2008). *J. Appl. Cryst.* **41**, 466–470.

[bb13] McNulty, J. & McLeod, D. (2011). *Synlett*, **5**, 717–721.

[bb14] Noland, W. E., Walhstrom, M. J., Konkel, M. J., Brigham, M. E., Trowbridge, A. G., Konkel, L. M. C., Gourneau, R. P., Scholten, C. A., Lee, N. H., Condoluci, J. J., Gac, T. S., Pour, M. M. & Radford, P. M. (1993). *J. Heterocycl. Chem.* **30**, 81–91.

[bb15] Rowland, R. S. & Taylor, R. (1996). *J. Phys. Chem.* **100**, 7384–7391.

[bb16] Sheldrick, G. M. (1996). *SADABS*. University of Göttingen, Germany.

[bb17] Sheldrick, G. M. (2008). *Acta Cryst.* A**64**, 112–122. 10.1107/S0108767307043930 18156677

[bb18] Sheldrick, G. M. (2015). *Acta Cryst.* C**71**, 3–8.

[bb19] Shiri, M., Zolfigol, M. A., Kruger, H. G. & Tanbakouchian, Z. (2010). *Chem. Rev.* **110**, 2250–2293. 10.1021/cr900195a 20041637

[bb20] Westrip, S. P. (2010). *J. Appl. Cryst.* **43**, 920–925.

[bb21] Zhang, W., Liu, Z., Li, S., Yang, T., Zhang, Q., Ma, L., Tian, X., Zhang, H., Huang, C., Zhang, S., Ju, J., Shen, Y. & Zhang, C. (2012). *Org. Lett.* **14**, 3364–3367. 10.1021/ol301343n 22694269

